# Metformin in the management of antipsychotic-induced weight gain in adults with psychosis: development of the first evidence-based guideline using GRADE methodology

**DOI:** 10.1136/ebmental-2021-300291

**Published:** 2021-09-29

**Authors:** Ita Fitzgerald, Jean O'Connell, Dolores Keating, Caroline Hynes, Stephen McWilliams, Erin K Crowley

**Affiliations:** 1 Pharmacy Department, Saint John of God Hospitaller Services, Dublin, Ireland; 2 School of Pharmacy, University College Cork, Cork, Ireland; 3 Endocrinology, St Columcille's Hospital, Loughlinstown, Ireland; 4 Endocrinology, St Vincent's University Hospital, Dublin, Ireland; 5 Pharmacy Department, Saint John of God Hospital, Dublin, Ireland; 6 Pharmacy, Saint John of God Hospitaller Services, Dublin, Ireland; 7 School of Medicine and Medical Sciences, University College Dublin, Dublin, Ireland; 8 Medical, Saint John of God Hospitaller Services, Dublin, Ireland; 9 Pharmaceutical Care Research Group, University College Cork, Cork, Ireland

**Keywords:** adult psychiatry, schizophrenia & psychotic disorders

## Abstract

**Background:**

Adjunctive metformin is the most well-studied intervention in the pharmacological management of antipsychotic-induced weight gain (AIWG). Although a relatively unaddressed area, among guidelines recommending consideration of metformin, prescribing information that would facilitate its applied use by clinicians, for example, provision of a dose titration schedule is absent. Moreover, recommendations differ regarding metformin’s place in the hierarchy of management options. Both represent significant barriers to the applied, evidence-based use of metformin for this indication.

**Objective:**

To produce a guideline solely dedicated to the optimised use of metformin in AIWG management, using internationally endorsed guideline methodology.

**Methods:**

A list of guideline key health questions (KHQs) was produced. It was agreed that individual recommendations would be ‘adopted or adapted’ from current guidelines and/or developed de novo, in the case of unanswered questions. A systematic literature review (2008–2020) was undertaken to identify published guidelines and supporting (or more recent) research evidence. Quality appraisal was undertaken using the Appraisal of Guidelines Research and Evaluation II tool, A Measurement Tool to Assess Systematic Reviews (AMSTAR) assessment, and the Cochrane Risk of Bias 2 tool, where appropriate. Assessment of evidence certainty and recommendation development was undertaken using Grading of Recommendations Assessment, Development and Evaluation (GRADE) methodology.

**Findings:**

We confirmed that no published guideline—of appropriate quality, solely dedicated to the use of metformin to manage AIWG was available. Recommendations located within other guidelines inadequately addressed our KHQs.

**Conclusion:**

All 11 recommendations and 7 supporting good practice developed here were formulated de novo.

**Clinical implications:**

These recommendations build on the number and quality of recommendations in this area, and facilitate the optimised use of metformin when managing AIWG.

## Background

### Antipsychotic-induced weight gain management: current standard of management

During the first years of antipsychotic treatment, approximately 80% of patients with first episode psychosis (FEP) gain a clinically significant amount of weight (>7% of their baseline body weight).^1 2^ In the case of most antipsychotics, time to plateau of antipsychotic-induced weight gain (AIWG) remains unknown or uncertain.^3^ AIWG is a particularly important side effect, as it mediates cardiometabolic outcomes, such as development of type 2 diabetes mellitus (T2DM) and subsequent cardiovascular disease—the latter being responsible for approximately 60% of the excess mortality among those with schizophrenia.^4^ AIWG also causes much psychological distress.^5–7^AIWG has been shown to negatively impact quality of life,^5 6^ and is a common cause of antipsychotic non-adherence and premature discontinuation.^7^ Despite its prevalence and impact, across Ireland and the UK, there is no care pathway or evidence-based intervention applied systematically when managing AIWG.^8^ Furthermore, no guideline exists that solely addresses AIWG management, although some recommendations can be found within larger guidelines. Most of these recommendations endorse the sequential use of lifestyle interventions, switching antipsychotic to a lower-risk agent, and subsequent consideration of adjunctive metformin.^9–11^ While pragmatic, application of this approach to successfully attenuate AIWG has not been studied empirically. Some interventions included in the hierarchical model also have been associated with non-significant anthropometric changes—primarily switching antipsychotics to attenuate AIWG.^12^ To add further complexity, other recommendations are relatively unspecific regarding a preferred management approach.^13 14^ Thus, conflicting, unspecific recommendations predominate this area and have likely contributed to the confusion, varying practices, and frequent clinician inertia surrounding AIWG management.^8 15^

### Metformin in AIWG management: a missed opportunity

Of all pharmacological interventions, metformin treatment is associated with the most consistent supporting evidence, having been assessed in several well-designed meta-analyses of randomised controlled trials (RCTs).^16 17^ However, adjunctive metformin treatment to manage AIWG remains infrequent and unsystematic.^8 15^ Aside from conflicting recommendations regarding when metformin should be considered, no prescribing information outlining how metformin should be used is provided for.^9–11 13 14^ Further research on metformin as a treatment intervention is unlikely to yield substantial changes in its recommended use in practice, aside from its role in preventing AIWG. In our opinion, given the scale of the problem and associated physical and psychological harms, current evidence gaps are not sufficient to preclude its more widespread use. To do this effectively, clinicians need guidance. This paper describes the process of developing the first guideline dedicated solely to the use of metformin in managing AIWG.

## Objectives

1. Quality assess available recommendations and determine to what extent evidence supporting metformin is incorporated into recommendations.
2. Identify whether there is a role for metformin as a first-line intervention—not solely where diet and lifestyle, or switching antipsychotics, have failed.
3. Assess the optimum prescribing parameters that facilitate metformin’s use in managing AIWG, including when and how it should be used.

## Methods

**Figure 1:**
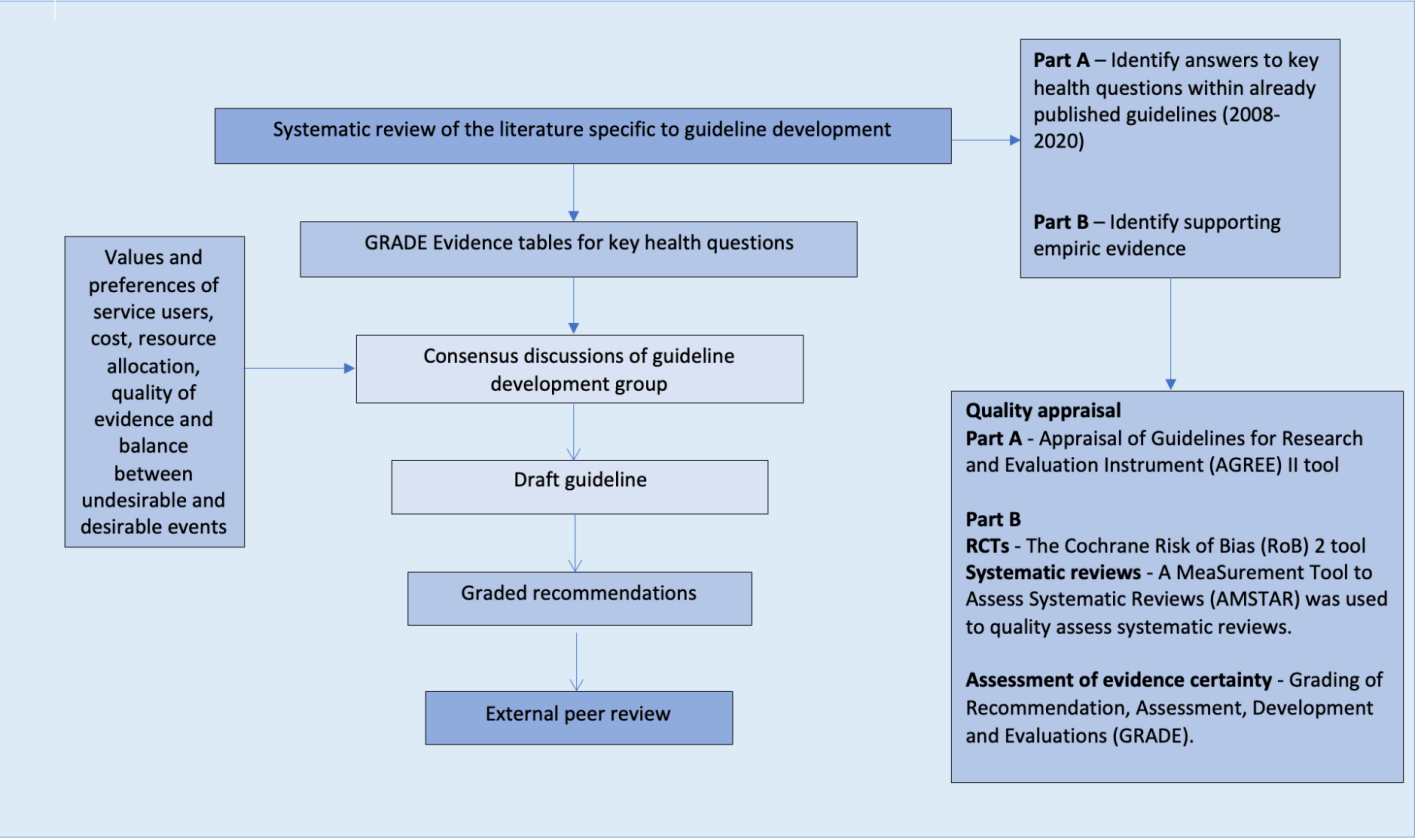
Summary of guideline development process. Created by authors. RCTs, randomised controlled trials.

An overview of the guideline development process can be found in figure 1. A more detailed summary is located in the online supplemental appendix. A list of Key Health Questions (KHQs) was developed, outlining all areas that guideline recommendations would address. It was agreed that where quality allowed, recommendations would be adapted or adopted from published recommendations. Where this was not possible, or in the case of unanswered KHQs, recommendations were formulated de novo.

10.1136/ebmental-2021-300291.supp1 Supplementary data

A systematic literature review is an essential first step when developing a guideline.^18^ However, the purpose of this paper is to discuss the process of moving from evidence to recommendations, to outline the full spectrum of recommendations developed, and the novelties that lie therein. While this paper will make frequent reference to its results, a more detailed description of the literature review is outlined in the online supplemental appendix. Particularly important findings, especially those that influenced choices to adapt current or develop de novo recommendations, will be discussed here. Due to resourcing, we relied on unsystematic reviews of literature and clinician experience as representation of patient preferences and values.

### Guideline development group

The guideline development group (GDG) consisted of consultant psychiatrists, specialist pharmacists and psychiatric nurses, as well as those experienced in project management and guideline development. No conflicts of interest were reported.

### Developing and grading recommendations

Following literature review completion, consensus meetings began. An informal consensus approach was chosen due to topic complexity and project resources. Consensus was defined as general group acceptance on direction and wording of all recommendations and good practice points. Whether to make a recommendation or good practice point, and the accompanying direction and strength of recommendations, were all decided on using the principles and processes of Grading of Recommendations Assessment, Development and Evaluation (GRADE).^18^ GRADE evidence profiles were developed and used to assign a quality of evidence rating for each KHQ addressed. Good practice points were used to provide guidance on important aspects of metformin prescribing where little evidence was available, or in cases where the actions highlighted were an obvious part of routine clinical care. The GDG agreed that recommendations would apply only to adults (18–65 years old) with psychotic illnesses. Recommendations would not address the role of metformin in preventing AIWG. External review of recommendations was undertaken by obesity management specialists from both endocrinology and psychology professions.

As per GRADE, recommendations can either be strong or weak (conditional). Their interpretations are contained in table 1.^18^

**Table 1:** GRADE system—strength of recommendation^18^

Recommendation strength	GDG consensus	Interpretation for clinicians
Strong recommendation	The GDG was confident that the desirable effects of adhering to the recommendation outweigh the undesirable effects	All or almost all individuals would want the recommended course of action, and only a small proportion would not; therefore.
Weak (conditional) recommendation	The GDG concluded that the desirable effects of adhering to the recommendation probably outweigh the undesirable effects, but the group is not confident about these trade-offs.	Most individuals would want the suggested course of action, but many would not.
In both cases, clinicians should recognise that different choices will be appropriate for individual patients, and that they must help each patient arrive at a management decision consistent with their values and preferences. In the case of a weak recommendation, variation in patient values and preferences are likely to be greater and therefore, healthcare providers need to devote more time to the process of shared decision making, by which they ensure that the informed choice reflects individual values and preferences.		

GDG, guideline development group; GRADE, Grading of Recommendations Assessment, Development and Evaluation.

**Figure 2:**
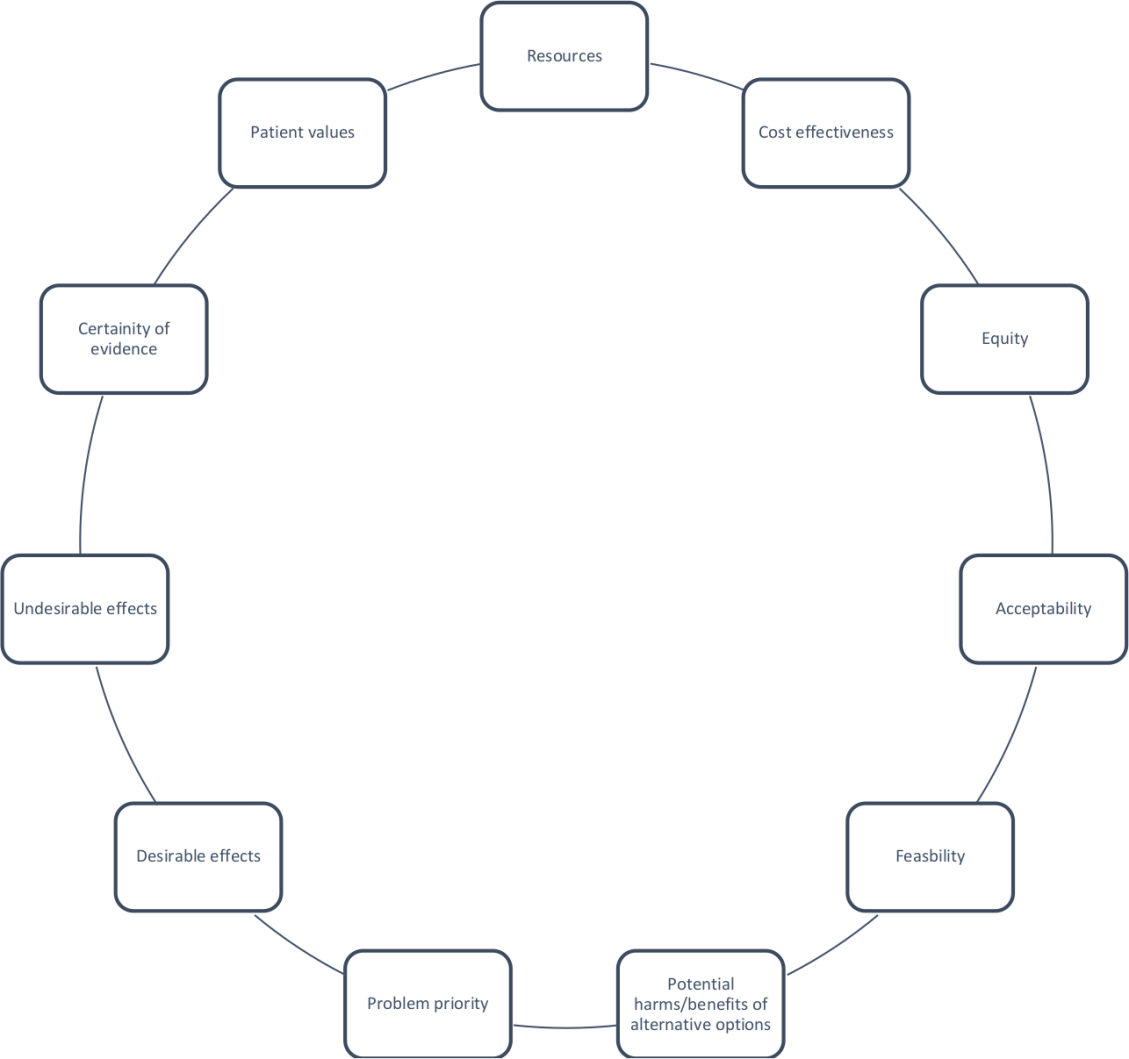
Variables for consideration when moving from evidence to recommendations. Created by authors.^18^

Recommendation development and assignment of recommendation strength is based on several factors, not solely quality of evidence.^18^ Other factors that were considered by the GDG are outlined in figure 2.

### Values and preferences of patients

Available research and extensive clinician experience, reflective of both psychiatry and endocrinology perspectives,^7 8 15^ highlighted that all or almost all patients place a high value on minimising current and avoiding further weight gain, and in such cases have not experienced refusal to consider taking an additional medication, typically due to the extent of physical and psychological adverse effects of AIWG. Member experience was that patients typically place a greater value on managing AIWG with metformin, compared with limiting their tablet burden and risk of transient gastrointestinal side effects. Patients also appreciate metformin’s low cost, its availability, and its ability to suppress increased appetite and cravings for high-energy foods typical of AIWG,^9^ as this is not addressed through diet and lifestyle interventions. Limited research on the preferences of patients on managing AIWG also demonstrated a willingness to take metformin, and that it was clinicians’ reluctance to prescribe metformin identified as a treatment barrier.^8 15^

## Findings

Detailed results of the systematic review, including all evidence from guidelines and supporting research evidence, along with their formal quality appraisal, are located in the online supplemental appendix.

### Appraisal of Guidelines Research and Evaluation (AGREE) II assessment of guideline recommendations

Although two guideline recommendations, produced by the WHO in 2018 and the Scottish Intercollegiate Guidelines Network (SIGN) in 2013, were judged as being of acceptable quality for adaptation, it was agreed that the SIGN recommendation had lost currency due to significant research on AIWG management methods published since.^12 16^ Appraisal of Guidelines Research and Evaluation (AGREE) II assessment of the WHO recommendation reflected a lack of evidence integration into their recommendation, which advocates for antipsychotic switching and/or adoption of lifestyle measures to have failed before metformin be considered.^10^ A 2019 meta-review assessing efficacy of all AIWG management strategies showed antipsychotic switching to be associated with non-significant effects on weight outcomes,^12^ as originally indicated in a 2010 Cochrane systematic review.^19^ Lifestyle interventions associated with the largest effect sizes are resource-intensive and include use of psychoeducational programmes, individual lifestyle counselling and supervision, alongside specialist dietician input.^12^ Their high incremental cost relative to their moderate short-term benefits demonstrated thus far makes their widescale adoption by patients and policy-makers challenging, and impossible in many settings. Considered collectively, it was decided to formulate de novo recommendations for all KHQs.

### Key health questions addressed

A brief overview of the empiric evidence addressing all KHQs is outlined below. A full overview of reasons for downgrading evidence quality are outlined in GRADE evidence tables contained in the online supplemental appendix.

#### KHQ 1: should metformin versus usual care or placebo be used in the management of AIWG in adults with established psychosis?

The most recent meta-analysis identified was a 2016 study by De Silva *et al*.^17^ Compared with placebo (n=340), metformin treatment (n=341) resulted in a mean reduction in weight −3.24 kg (95% CI −4.72 to −1.76) (p<0.001) and body mass index (BMI) −1.11 (95% CI −1.62 to −0.60) (p<0.001). The associated I^2^=85% (p<0.001). Subgroup analysis identified most heterogeneity was due to pooling of FEP and chronic participants (discussed further under KHQ 4).^17^ As time to plateau is unknown in the case of many antipsychotics, but has been cited as taking months (olanzapine) and years (clozapine) to occur,^2 3^ potential induction of a plateau of AIWG among study participants must also be acknowledged. Limitations of the studies assessed included^17^:

- Short follow-up period (mean duration of 16 weeks, range 12–24 weeks).
- Uneven distribution of lifestyle interventions across RCTs.
- No grey or unpublished literature included—associated funnel plot found no evidence of publication bias.

A single RCT published after this meta-analysis by Chiu *et al*, (placebo, n=19; metformin 500 mg, n=18; metformin 1000 mg, n=19) explored the impact of metformin dosing on anthropometric outcomes over 12 weeks. They found metformin 1000 mg treatment to be associated with significantly greater weight loss compared with baseline (p<0.05),^20^ but was associated with less weight loss overall when indirectly compared with results of the De Silva *et al*, meta-analysis.^17^ Those assigned to metformin 1000 mg on average lost 1 kg (p<0.05), whereas those assigned to metformin 500 mg or placebo lost non-signficant amounts of weight after 12 weeks treatment. Those assigned to both metformin groups reported significant reductions in BMI, with a mean decrease of 0.70 kg/m^2^ (p=0.021) and 0.50 kg/m^2^ (p=0.017) in those assigned to metformin 500 mg and 1000 mg, respectively.^20^ Differences in BMI outcomes between the two groups, if replicable, were not considered clinically insignificant. No measures of variance were provided alongisde any point estimates and requests for same from the authors went unanswered.^20^ Potential reasons for lesser comparative anthropometric changes to those previously reported may be the low dose used (discussed further under KHQ 5), and participants being those with significant prior antispychotic exposure (discussed further under KHQ 4).^20^

GRADE quality of evidence rating for this KHQ was moderate for all anthropometric outcomes due to unclear risk of bias in a minority of studies included in the De Silva *et al*,^17^ meta-analysis and evidence of inconsistency in the RCT by Chiu *et al*.^20^ Quality of evidence overall was considered low for the adverse event outcome. Although rated as high for the Chiu *et al* RCT,^20^ in the meta analysis, there was evidence of selective and incomplete outcome reporting in some studies included. Although data were not pooled due primairly to missing data and small event numbers, results appeared inconsistent across studies assessing adverse effects, and thus, evidence quality was also downgraded due to inconsistency.^17^

#### KHQ 2: should metformin versus non-pharmacological methods be used in the management of AIWG?

and

#### KHQ 3: should metformin + non-pharmacological methods versus non-pharmacological methods be used in the management of AIWG?

One RCT that directly compared metformin to a combination non-pharmacological intervention was identified. This intervention involved psychoeducational, dietary and exercise components. As in table 2, results showed metformin to be more effective than placebo—both alone and in combination with the non-pharmacological intervention (p<0.001).^21^

**Table 2:** Summary of evidence addressing efficacy of metformin and a diet and lifestyle intervention^21^

Group/outcome	Placebo (n=32)	Metformin (750 mg/day)+non-pharmacological intervention (n=32)	Metformin (750 mg/day) alone (n=32)	Non-pharmacological intervention alone (n=32)
Mean change in weight (kg)	3.1 (95% CI 2.4 to 3.8)	−4.7 (95% CI −3.4 to −5.7)	−3.2 (95% CI −2.5 to −3.9)	−1.4 (95% CI −0.7 to −2.0)

Evidence quality was moderate across all outcomes for this KHQ. Downgrading was due to indirectness, as participants only included those with FEP.^21^ No study replicating these results was identified. A 2019 meta-review showed indirect evidence that individual lifestyle counselling was associated with a marginally better mean weight reduction, compared with both metformin and group lifestyle coaching (p<0.001). Participants studied included those with chronic and FEP illnesses.^12^ Evidence for switching antipsychotics as a safe and effective means to manage AIWG, as discussed under section 3.1, was also appraised here.^12 19^ The GDG agreed not to endorse antipsychotic switching as a failed strategy prior to considering metformin, due to lack of supporting evidence of effective weight reversal and considerable associated risk.^12 19^

#### KHQ 4: should metformin versus usual care or placebo be used in the treatment of AIWG in adults with FEP?

Significant heterogeneity in the De Silva *et al,* meta-analysis was subsequently identified to be due to pooling of FEP and chronic psychosis participants. When assessed separately, significant between-group differences were seen (p<0.001). The associated I^2^ result was 0% (p=0.59) in FEP participants and 11% (p=0.35) in chronic psychosis participants. FEP participants (n=283) mean change in weight compared with placebo was −5.94 kg (95% CI −6.75 to −5.12) (p<0.001). Mean weight change among those with chronic psychosis (n=460) was −2.06 kg (95% CI −2.71 to −1.41) (p<0.001).^17^ For change in weight, evidence quality was moderate. Downgrading occurred due to high risk of bias in one of five studies included, and an unclear risk of bias in two others. Adverse event data was not reported separately for each group.^17^

#### KHQ 5: where metformin is identified as being effective in a particular cohort, what dose of metformin should be used?

A range of doses between 500–2000 mg/day was applied across studies. The median dose was 1000 mg/day (IQR=1000 mg/day).^17 20^ Formal dose-response data wasn’t identified.

#### KHQ 6: where metformin is identified as being effective in a particular cohort, for how long should metformin be used?

Median trial length was 12 weeks (range 12–24 weeks).^17 20^ One study assessing effects of discontinuing metformin in those with a previous positive response was identified, which showed beneficial effects being lost with time following discontinuation.^22^

#### KHQ 7: where metformin is being used for the treatment of AIWG versus usual care or placebo, what are the potential harms associated with its use in adults with psychosis?

Five out of 10 adult studies (metformin, n=215; placebo, n=211) in the De Silva *et al*, meta-analysis reported data on discontinuation rates.^17^ In all studies, there was no signficant difference in discontinuation rates between placebo and metformin despite some studies reporting numerical differences in rates of adverse effects, highlighting the mild and transient nature of the most common adverse event associated with metformin treatment—gastrointestinal side effects. Eight out of 10 of studies (metformin, n=292; placebo, n=287) did report adverse event data, primairly relating to the gastrointestinal tract. None reported serious adverse effects due to metformin. Only six out of the 8 studies reported whether there was a significant difference in adverse events between groups, with only one reporting diarrhoea was significantly more common in the metformin group (metformin 33% vs placebo 19%, p=0.018).^17^ Chiu *et al*, reported no significant diffences in adverse events or discontinuation rates between groups, and reported no serious adverse events in any group.^20^ GRADE quality of evidence rating was low for this outcome due to evidence of selective and incomplete outcome reporting in the De Silva *et al*, meta analysis.^17^

### De novo recommendations

The following boxes 1 and 2 contain all recommendations developed and externally reviewed. Expansion on the rationale, particularly where a ‘strong’ recommendation strength was issued in the absence supporting high quality evidence, will be provided for in the discussion. Note that recommendations herein only apply to non-diabetic populations. In those who have a diagnosis of T2DM, and have experienced distressing AIWG for which pharmacological management may be suitable, optimisation of metformin prescribing may be considered, or further pharmacological treatment with a glucagon-like peptide-1 agonist, for example, semaglutide, where the evidence base in T2DM is now substantial,^23^ and in AIWG treatment emerging.^24^

Box 1 **Recommendations developed addressing the point at which metformin should be considered for antipsychotic-induced weight gain (AIWG) management and associated baseline screening**

**Area 1 (KHQ 1–4): appropriateness of metformin**

**Recommendation 1**: The use of metformin in the treatment of AIWG can be applied in two ways; as part of an early intervention strategy or in the treatment of established weight gain. We recommend that preference should be given to early intervention strategies, where possible.

*Strength of recommendation:* Strong. *Quality of evidence*:Low

**Recommendation 2**: For the purposes of this guideline, early intervention in the management of AIWG is defined as the implementation of an intervention following a ≥7% increase in baseline body weight, within 1 month of antipsychotic treatment. *(good practice point)*

**Recommendation 3**: In the case of either early intervention or treatment, where non-pharmacological interventions are deemed appropriate and acceptable to the patient we recommend that these be offered before metformin.

*Strength of recommendation*: Strong *Quality of evidence*: Moderate

**Recommendation 4**: Where lifestyle interventions available to patients are unacceptable to them or are inappropriate, for example in the case of physical disability, we recommend the use of metformin as an alternative first-line intervention.

*Strength of recommendation:* Strong *Quality of evidence*: Moderate

**Recommendation 5:** Where non-pharmacological interventions are appropriate but seemingly ineffective, we recommend metformin be offered as an alternative. It should be noted that evidence supports improved efficacy of metformin in attenuating AIWG when initiated at earlier time points in antipsychotic treatment. Therefore, what constitutes an appropriate trial length of non-pharmacological interventions must consider this.

*Strength of recommendation:* Strong *Quality of evidence*: Moderate

**Recommendation 6**: We recommend the use of metformin to attenuate weight gain induced by any antipsychotic.

*Strength of recommendation*: Strong *Quality of evidence:* Moderate

**Area 2 (KHQ 7): Initiating metformin (baseline screening)**

Recommendation 1: Baseline renal function must be assessed before treatment is started. Where the Estimated Glomerular Filtration Rate (eGFR) is <60 mL/min, dosing of metformin should be adjusted. Metformin is contraindicated in those with an eGFR of <30 mL/min. (*good practice point*)

Box 2 **Recommendations developed addressing optimal metformin dosing, proosed treatment goals, ongoing monitoring and management of side effects, alongside deprescribing**

**Area 3 (KHQ 5): metformin dosing**

**Recommendation 1:** We recommend metformin be started at 500 mg twice daily with meals. Metformin dosing should be increased in increments of 500 mg every 1–2 weeks.

*Strength of recommendation*: Strong *Quality of evidence*: Low

**Recommendation 2**: We recommend a target dose of metformin of 2000 mg/day. The target dose, however, should consider individual tolerability and evidence of efficacy.

*Strength of recommendation:* Strong *Quality of evidence:* Moderate

**Area 4 (KHQ 6): Assessing response to treatment**

**Recommendation 1**: If metformin is being used as part of an early intervention strategy, we recommend that plateau of weight gain should be the goal of treatment. Reversal of weight gained to date due to antipsychotic treatment may also be feasible.

*Strength of recommendation:* Strong *Quality of evidence:* Moderate

**Recommendation 2:** Where metformin is being used to induce weight loss in those with established antipsychotic-induced weight gain, we suggest the goal of metformin treatment be to induce a weight loss of at least 5% of baseline body weight within 6 months of treatment.

*Strength of recommendation*: Conditional *Quality of evidence*: Low

**Recommendation 3:** Goals of treatment should be individualised and agreed collaboratively with the patient. *(good practice point)*

**Area 5 (KHQ 7): Ongoing monitoring**

**Recommendation 1:** Renal function should be monitored annually. In those who are at increased risk of renal impairment for example, those with chronic kidney disease or the elderly, renal function should be measured every 3–6 months. *(good practice point)*

**Recommendation 2:** Intermittent monitoring of vitamin B_12_ levels is recommended, especially where evidence of megaloblastic anaemia is present. *(good practice point)*

**Recommendation 3**: Clinicians should also monitor the patients’ adherence and tolerability to both the antipsychotic and the metformin regularly. *(good practice point)*

**Area 6 (KHQ 7): Management of side effects**

**Recommendation 1:** Gastrointestinal side effects are dose related and can be managed through dose reduction and/or a slower dose titration. *(good practice point)*

**Recommendation 2:** The estimated incidence of lactic acidosis is 4.3 per 100 000 person-years in metformin users. Adjustment of dose to account for low levels of renal function will help to mitigate risk. Additionally, avoidance of metformin in certain groups—including those with a history of alcohol misuse or in those who are prescribed interacting medicines will also reduce risk of lactic acidosis occurring. *(good practice point)*

**Area 7 (KHQ 6): Deprescribing**

**Recommendation 1:** Where treatment goals have been reached at 6 months, we recommend metformin be continued. However, lack of evidence to support the continuation of metformin beyond 6 months must be considered as part of the risk–benefit assessment.

*Strength of recommendation:* Strong. *Quality of evidence:* Low

**Recommendation 2:** Where agreed treatment goals have not been reached at 6 months, we recommend that treatment be reviewed and the following undertaken:

- The dose of metformin should be increased to 2000 mg/day, where possible.
- If treatment has been optimised as much as possible, treatment should be stopped
- Clinicians should check adherence, and stop if not mostly adherent.

*Strength of recommendation*: Strong. *Quality of evidence:* Low

## Discussion

This work represents an approach to optimise use of a modestly effective AIWG management strategy,^12^ and suggests novel applications of its use to improve patient-important outcomes. The systematic review and quality assessment of previously published recommendations identified areas of ambiguity, suboptimal evidence integration, and unanswered questions as to when and how metformin should be used to manage AIWG.^9–14^ Such gaps were subsequently addressed here.

### Assessing evidence integration across current recommendations

While direct comparisons are largely lacking, indirect evidence shows individualised lifestyle interventions to be associated with the largest comparative effect size for weight reduction, alongside a moderate effect size in BMI and waist circumference reduction. Group lifestyle interventions the current typical standard of delivery, were associated with a small effect size on weight and BMI reduction Metformin, alone and in combination with lifestyle interventions, was associated with a moderate effect size on weight reduction. Switching antipsychotics to attenuate AIWG was associated with non-significant effect sizes.^12^ Thus, published recommendations are now largely outdated and do not reflect accruing evidence in this area.^9–14^ ‘Lifestyle interventions’ currently endorsed as the preferred first-line approach are heterogeneous by definition. Furthermore, their replication and implementation needs to be considered in the context of the complex environments in which they will be delivered. RCT settings, participants included and resources required to deliver individualised interventions are in many cases not reflective of standard clinical practice.^10^ Considering the frequency and burden of AIWG,^25^ sustainable change must be led by scalable interventions. Thus, while engaging with individualised, tailored lifestyle interventions should be considered the gold standard, current resourcing means that moderately effective group-based interventions delivered to a much broader cohort likely represents the most efficient vehicle of to produce widespread change within this category of interventions

### Metformin as a first-line strategy

For a significant proportion of patients, uptake of any lifestyle intervention will be refused, inappropriate at the time of offering or ineffective.^8 15^ Without intervention, AIWG can occur rapidly, with the largest proportion of total weight gained occurring within the first year.^1^ Metformin offers a safe and similarly effective intervention to many lifestyle approaches, with superior efficacy to switching antipsychotics,^12^ but with much lesser associated risk. Metformin use as part of a first-line intervention is not addressed via current recommendations.^9–11 13 14^ In systematic reviews and subsequent meta-analyses of RCTs, the focus is always on metformin’s effect on weight reversal.^16 17^ However, for many, this result also includes induction of a plateau of AIWG.^2 3^ Metformin is likely to be more effective in attenuating AIWG before onset of significant insulin resistance and thus, demonstrates greater efficacy among those with a lower antipsychotic burden.^17^ Earlier initiation is likely to maximise potential results and minimise overall weight gain, where the greatest potential benefits are through early induction of a plateau of AIWG. Metformin may also positively influence patients overall cardiometabolic risk profile, including improved blood glucose control.^12^ The GDG issued a strong recommendation in support of metformin as an alternative first-line strategy where diet and lifestyle interventions are ineffective, inappropriate or unacceptable. Aside from evidence on comparative effectiveness with other interventions, this decision considered the following^4 8 12 16 17 26 27^:

- Psychological impact of AIWG.
- Prevalence of AIWG and its significant contribution to obesity rates in schizophrenia.
- Ease of recommendation implementation.
- Raised standardised mortality ratio compared with the general population, including pre-antipsychotic.
- Low cost and associated resource use.
- Established long-term safety profile in T2DM.
- Very rare likelihood of catastrophic harm.
- Current lack of other pharmacological methods with a similar cost, safety and risk profile.
- Objectivity of weight outcomes.
- Potential for improvement in quality of care.
- Equitable access across socioeconomic groups compared with effective diet and lifestyle interventions.
- No interaction with the cytochrome P450 system and therefore, minimal pharmacokinetic drug interactions—there are no known interactions with any antipsychotics or other psychotropics, including mood stabilisers, antidepressants and antianxiety medications.

To encourage prompt action, the GDG provided a definition of what constituted an ‘early intervention’, highlighted what is considered clinically significant AIWG, and introduced a new goal of treatment—early plateau of AIWG.

### Strong recommendations in the absence of high-quality evidence

Some recommendations were rated as ‘strong’ in the absence of supporting high-quality evidence. As per GRADE, recommendations strength depends in part—not solely on the level of confidence in the intervention effects.^18^ GRADE highlights several examples where issuing a strong recommendation in the case of lower quality supporting evidence is appropriate.^18 28^ For example, in the case of potentially serious threats to health, or where potentially equivalent options are available, but one is clearly less risky or costly than the other. In both cases, a high value is placed on avoiding harm.^28^ Where a strong recommendation was issued in the absence of equivalent level evidence, the GDG considered the social, economic and personal impact of AIWG. Subsequent recommendations reflect a belief that all or almost all patients place a relatively low value on the additional pill burden, increased risk of transient gastrointestinal side effects and very small absolute increased risk of lactic acidosis, and place a high value on minimising further, or reducing current, AIWG. It was agreed that the endorsement of those recommendations with a strong rating was congruent with GRADE guidance,^18 28^ and like other recommendations made by international GDG, including the WHO.^29 30^ Furthermore, although we did not consider guidelines that addressed general obesity management due to reasons outlined, recommendations outlined here are considered congruent with national public health guidance principles on obesity management in Ireland,^31^ and guidance on prevention of T2DM in high risk groups in the UK.^32^

### Moving forward

A comprehensive review of AIWG management is well overdue. Replication of a similar hierarchical model applied in the general population does not account for the unique challenges faced by those with psychotic illnesses, including the disproportionate numbers of risk factors present for becoming overweight or obese.^25^ Extensive interindividual variability exists regarding the burden and pattern of weight gained following antipsychotic initiation.^1–3^ Different antipsychotics also present with markedly different risks of inducing clinically significant AIWG.^3^ Thus, AIWG management pathways must reflect not only the underlying evidence base, but also a range of risk profiles associated with this side effect. Although pragmatic, a tiered approach to management and endorsement of a ‘one-size-fits-all’ approach to AIWG management algorithms is not appropriate. This was recently highlighted by guidance produced in the UK in managing obesity in secure mental health settings, where the need for a tailored approach was highlighted.^33^ A more suitable approach may be represented by a series of management pathways, stratified according to risk. Clearly, there is much work to be done before a more general guideline for managing AIWG can be produced, that accounts not only for the changing evidence base in management methods, but also for the intricacies and nuances associated with AIWG presentation. We are hopeful the work contained here could be easily integrated into such a body of work.

To date, interventions have focused on what patients should do to reduce their weight. In contrast possible routes for clinicians and psychiatric services to make changes have been neglected. This includes improving access to medications that manage weight and other cardiometabolic risk factors. Set against its clear and unarguable effects, negative associations with metformin become relatively less important. On balance, coprescription of metformin to a much wider range of patients is evidently desirable.^34^

### Limitations

Limitations of metformin as an intervention have been discussed in detail elsewhere.^12 16 17^ One potential practical limitation of recommendation implementation is the ‘off label’ use of metformin, as it is not licensed for this indication in Ireland or the UK. The ‘off label’ use of medications in the pharmacological management of AIWG is likely to be a pertinent issue among clinicians and policy makers. Currently, there are two licensed antiobesity agents for use in the general population—orlistat, which has been shown to be associated with non-significant effects on any physical health outcome when studied among those with schizophrenia,^12^ and liraglutide, where the evidence base in AIWG managing is in its infancy and significant cost due to patent protection currently precludes widespread use. As there is minimal financial benefit to pharmaceutical companies in licensing metformin for this indication, publication of guidance outlined here to support the systematic and evidence-based use of metformin is likely to be particularly valuable to busy clinicians. Finally, these recommendations need to be assessed as to whether they result in sustainable change, particularly regarding metformin’s use as part of an early intervention strategy.

### Clinical implications

This work represents the first guideline solely dedicated to the use of metformin to manage AIWG, and builds on both the quality and number of recommendations available. Our view is that this research represents a significant step forward towards improving the application of an inexpensive and well-studied management method.

## Data Availability

Data sharing not applicable as no datasets generated and/or analysed for this study.
