# Supplementary material for: Metformin in the management of antipsychotic-induced weight gain in adults with psychosis: development of the first evidence-based guideline using GRADE methodology
Source: Evid Based Ment Health. 2021 Sep 29;25(1):15–22. doi: 10.1136/ebmental-2021-300291 (PMC8788031; doi:10.1136/ebmental-2021-300291)
Supplement: Supplementary data [file ebmental-2021-300291supp001.pdf]

Table of Contents

Part A - International guideline review 2

A.1 Search strategy outline 2

A.2 Inclusion and exclusion criteria 3

Part B - Empiric evidence review 5

B.1 Search strategy outline 5

B.2 Inclusion and exclusion criteria 6

Part C - International Guideline Review Results 7

C.1 Guideline repository search 7

C.2 Google search (1/2) 8

C.3 Google search (2/2) 9

C.4 Database search 10

Part D – PRISMA flow diagram of Literature review results (Part A) 12

Part E – AGREE 2 application to available guideline recommendations 13

Part F – Part B empiric evidence review results 14

PubMed, PsycINFO and Embase search results 14

Part G – PRISMA flow diagram of Literature review results (Part B) 15

Part H – Summary details of all Randomised Controlled Trials that met inclusion criteria (Part B) 16

Part I – GRADE evidence profiles 20

I.1 Table 1 - Should Metformin vs. Usual Care or Placebo be used in the Treatment of AIWG in Adults with Established Psychosis? 20

I.2 Table 2 – Should Metformin vs. Non-Pharmacological Treatment be used in the Management of AIWG with Psychosis? 25

I.3 Table 3 - Should Metformin + Non-Pharmacological treatment versus Non-Pharmacological treatment alone be used in the Management of AIWGs in Adults with Psychosis? 27

I.4 Table 4 - Should Metformin compared to Placebo or Usual Care be Used in the Management of AIWG in FEP? 30

Fitzgerald I, *et al. Evid Based Ment Health* 2022; 25:15–22. doi: 10.1136/ebmental-2021-300291

Part A - International guideline review

A.1 Search strategy outline

To be as inclusive as possible, a broad search strategy containing three subsections was designed;

1. Search of guideline developers websites, including specialist mental health sites:

    a) National Guideline Clearinghouse (NGC)
    b) Guidelines International Network (G-I-N)
    c) National Institute for Health and Clinical Effectiveness (NICE)
    d) Scottish Intercollegiate Guidelines Network (SIGN)
    e) Canadian Medical Association Infobase;
    f) Food and Drug Administration (FDA)
    g) British Association for Psychopharmacology (BAP)
    h) World Health Organization (WHO)
    i) The Royal Australian and New Zealand College of Psychiatrists (RANZCP)

2. Search of both general and subject-specific electronic journals including:

    a) PubMed
    b) Embase
    c) PsycINFO

For both the guideline and database search, several searches were undertaken using different combinations of MeSH terms and free text words, as outlined in Table 1. The search was carried out between December 2019 – January 2020.

Search number	Terms used
**1**	Antipsychotic OR Psychosis OR Schizophren* OR "Severe Mental Illness" AND Pharmac* OR Metformin OR Treatment OR Intervent* AND management AND "metabolic side-effects" OR "metabolic disturbance" OR "physical health"
**2**	Search 1 but substitute antipsychotic with psychotropic OR Neuroleptic OR Aripiprazole OR misulpride OR Chlorpromazine OR Clozapine OR Fluphenazine OR Haloperidol OR Olanzapine OR Risperidone OR Paliperidone OR Quetiapine OR Zuclopenthixol
**3**	Search 1 + 2 but substitute Metabolic side-effects/metabolic disturbances with "weight-gain" OR Weight

Table 1- Search strategy applied during guideline and database search

For the database search, a search date between 1/1/2008 -1/1/20 was applied to all searches following a preliminary review of the area identifying that the majority of research was published during this interval. This was complemented by a manual search of included guidelines' reference lists.

3. An advanced Google search, as guideline developers are increasingly publishing their guidelines on the Web for availability.

Google search criteria are outlined in Table 2. A search date of 1/1/2008 -1/1/2020 was applied and English language restriction set. The first 200 hits were screened. The search was carried out between December 2019 – January 2020.

Database	Search strategy
Google	1. Advanced search: "psychosis" AND this exact word or phrase "management in adults" + "guideline".<br><br>2. Advanced search: antipsychotic-induced weight gain AND this exact word or phrase "management in adults" + "guideline".

Table 2 - Criteria applied to the guideline advanced google search

A.2 Inclusion and exclusion criteria

Guidelines were **included** if they:

1. Were written in English;
2. Contained a focused section on pharmacological management of antipsychotic-induced weight gain (AIWG);
3. Were published between 1/1/2008 - 1/1/2020;
4. Specified the target population as adults ≥18 with a psychotic illness, other than in the context of Bipolar Affective Disorder (BPAD);
5. Are evidence-based guidelines i.e. those that include a report on systematic literature searches and contain explicit links between individual recommendations and supporting evidence and;
6. Were developed for use within countries with a relevant healthcare system for the Irish context.

Guidelines were **excluded** if they:

1. Didn't address any of our key health questions (KHQs);
2. Were developed in a healthcare system not applicable to the Irish setting;
3. Were written by only a single author;

4. Didn't contain a documented systematic evidence compilation and review process and;
5. Were guidelines based on obesity management in the general population only.

All guidelines were initially identified by their title from the three sources by one reviewer (IF). Two reviewers (IF and EC) then completed the second screen of all guidelines and article abstracts to identify those for full review. Differences were resolved via discussion. Data abstraction was undertaken by one reviewer (IF), but was then checked by a second reviewer (EC).

Part B - Empiric evidence review

B.1 Search strategy outline

The electronic database search included:

1. PubMed
2. Embase
3. PsycINFO
4. Cochrane Library (CENTRAL and database of systematic reviews)

Keywords chosen and associated synonyms are outlined in Table 3. A search date of between 1/1/2008 - 1/1/2020 was chosen. For all searches a search limit for "clinical trials" and an English language restriction was applied. Age was set to adult. The search was carried out between December 2019 – January 2020.

**Search number**	**Search terms used**
1	"Severe Mental Illness" OR schizophre* OR Psychosis OR Antipsychotic OR exp Psychotic Disorders OR First "Episode Psych*" AND Pharmac* OR Treatment OR Metformin OR Drug Therapy OR Manag* And Weight OR BMI OR Fat OR Manag* or Metabolic side effects OR Waist circumference
2	S1 but substitute antipsychotic with psychotropic* OR Neuroleptic OR Aripiprazole OR amisulpride OR asenapine OR Chlorpromazine OR Clozapine OR Fluphenazine OR Fluperazine OR Haloperidol OR Olanzapine OR Risperidone OR Paliperidone OR Quetiapine OR Zuclopenthixol

Table 3 - Outline of search number and terms used during empiric evidence search

The electronic database search was complemented by a manual search of reference lists. Unpublished sources or grey literature weren't included due to time and resource constraints. Published articles suggested by GDG members and not retrieved by systematic searching were also considered.

B.2 Inclusion and exclusion criteria

Evidence was **included** if:

i. Was based on Randomised Controlled Trials (RCTs), Cochrane or systematic reviews, or meta-analyses;
ii. Was published between 1/1/2008 - 1/1/2020;
iii. Was available in full text;
iv. Was published in a peer-reviewed journal;
v. Was published in English;
vi. The cohort were adults ≥18 with a psychotic illness, other than in the context of bipolar affective disorder;
vii. The intervention was the use of metformin alone in the treatment of AIWG;
viii. The comparator was placebo, usual care or non-pharmacological methods of treating AIWG and;
ix. Anthropometric measurements associated with the interventions(s) were reported as the primary outcome.

Evidence was **excluded** if:

i. The intervention was used to attenuate weight gain from other psychotropics, apart from antipsychotics;
ii. The applied use of metformin was solely in the prevention of AIWG;
iii. Was based on low levels of evidence including case reports, editorials and commentaries and;
iv. Primary outcomes reported weren't associated with anthropometric measurements.

Title review was undertaken by the primary author. Studies that clearly weren't relevant were eliminated. Abstracts of remaining studies were then eliminated by two independent reviewers. Effort was made to contact authors for clarification, where needed. Where uncertainty remained, third party consultation was available. Data extraction was primarily performed by the primary author using a pre-designed data extraction form.

Fitzgerald I, *et al. Evid Based Ment Health* 2022; 25:15–22. doi: 10.1136/ebmental-2021-300291

Part C - International Guideline Review Results

C.1 Guideline repository search

Resource name	URL	Category/Sub-category	Records as per inclusion/exclusion criteria
**Guidelines International Network (G-I-N)**	https://g-i-n.net/home	Library and resources/International Guideline Library	0
**National Institute for Health and Clinical Effectiveness (NICE)**	https://www.nice.org.uk	NICE guidance	1
**Scottish Intercollegiate Guidelines Network (SIGN)**	https://www.sign.ac.uk	Our guidelines	1
**CMA Infobase Clinical Practice Guidelines Database (CPGs)**	https://joulecma.ca/cpg/homepage	Guidelines and technology assessments	0
**Cochrane Schizophrenia group**	https://schizophrenia.cochrane.org	Resources	0
**Food and Drug Administration (FDA)**	https://www.fda.gov/drugs	Drugs/Guidance, Compliance, & Regulatory Information/Guidances (Drugs)	0
**British Association for Psychopharmacology (BAP)**	https://bap.org.uk	Publications/BAP Guidelines	1
**World Health Organization**	https://www.who.int	Health topics/Mental Health/Evidence and	1

**(WHO)**		Research	
**The Royal Australian and New Zealand College of Psychiatrists (RANZCP)**	https://www.ranzcp.org/practice-education/guidelines-and-resources-for-practice	Clinical practice guidelines/schizophrenia	2
**World Federation of Societies of Biological Psychiatry (WFSBP) Guidelines for Biological Treatment of Schizophrenia, Part 2: Update 2012 on the long-term treatment of schizophrenia and management of antipsychotic-induced side effects**	https://www.wfsbp.org/home/	WFSPB Treatment Guidelines and Consensus Papers	1
**National Health Service (NHS) (UK)**	https://www.evidence.nhs.uk/	Guidelines	2
**The Agency for Healthcare Research and Quality (AHRQ)**	https://www.ahrq.gov	Guidelines	0
**Total number of guidelines for health question screening**			9

C.2 Google search (1/2)

Search engine	Search term(s)	Date of search	Screened	Outputs
**Google**	Advanced search All these words: antipsychotic-induced weight gain AND this exact word or phrase "management in adults" "guideline" Limited to: English Language	22/12/2019	All 80 hits	Some repetition with guidelines already identified via guideline repository search.
				All 80 disregarded as not relevant for many reasons. These included documents identifying as: audit reports with accompanying recommendations based on other available national/international guidelines; obesity-based policy documents/books; guidelines not created for the target population/setting and thesis submissions.
**Total number of guidelines for health question screening**				0

C.3 Google search (2/2)

A second google search was undertaken to identify whether there were any guidelines based on psychosis management that contained a dedicated section related to the management of metabolic side effects of antipsychotics, namely antipsychotic-induced weight gain.

Google searched 22/09/2019

Search engine	Search term(s)	Date of search	Screened	Outputs
**Google**	Advanced search All these words: "psychosis" AND this exact word or phrase "management in adults" "guideline" Limited to: English Language	2/1/2020	All 80 hits	Some repetition with guidelines already identified via guideline repository search.<br>Other guidelines identified in the area of psychosis or schizophrenia but these did not contain a dedicated section on the pharmacological management of AIWG.
**Total number of guidelines for health question screening**				0

C.4 Database search

Database searches for existing guidelines

Search number	Terms used	Total hits across three databases
**1**	Antipsychotic OR Psychosis OR Schizophren* OR "Severe Mental Illness" AND Pharmac* OR Metformin OR Treatment OR Intervent* or AND management AND "metabolic side-effects" OR "metabolic disturbance" OR "physical health"	PubMed = 11<br>Embase = 0<br>PsychInfo= 0
**2**	Search 1 but substitute antipsychotic with psychotropic OR Neuroleptic OR Aripiprazole OR amisulpride OR Chlorpromazine OR	PsychInfo = 18<br>Embase = 4<br>PubMed = 43

	Clozapine OR Fluphenazine OR Haloperidol OR Olanzapine OR Risperidone OR Paliperidone OR Quetiapine OR Zuclopenthixol	
**3**	Search 1 + 2 but substitute Metabolic side-effects/metabolic disturbances with "weight-gain" OR Weight	PsychInfo = 19<br>Embase = 4<br>PubMed = 17
**Total used for title/executive summary review**		**0**

Part D – PRISMA flow diagram of Literature review results (Part A)

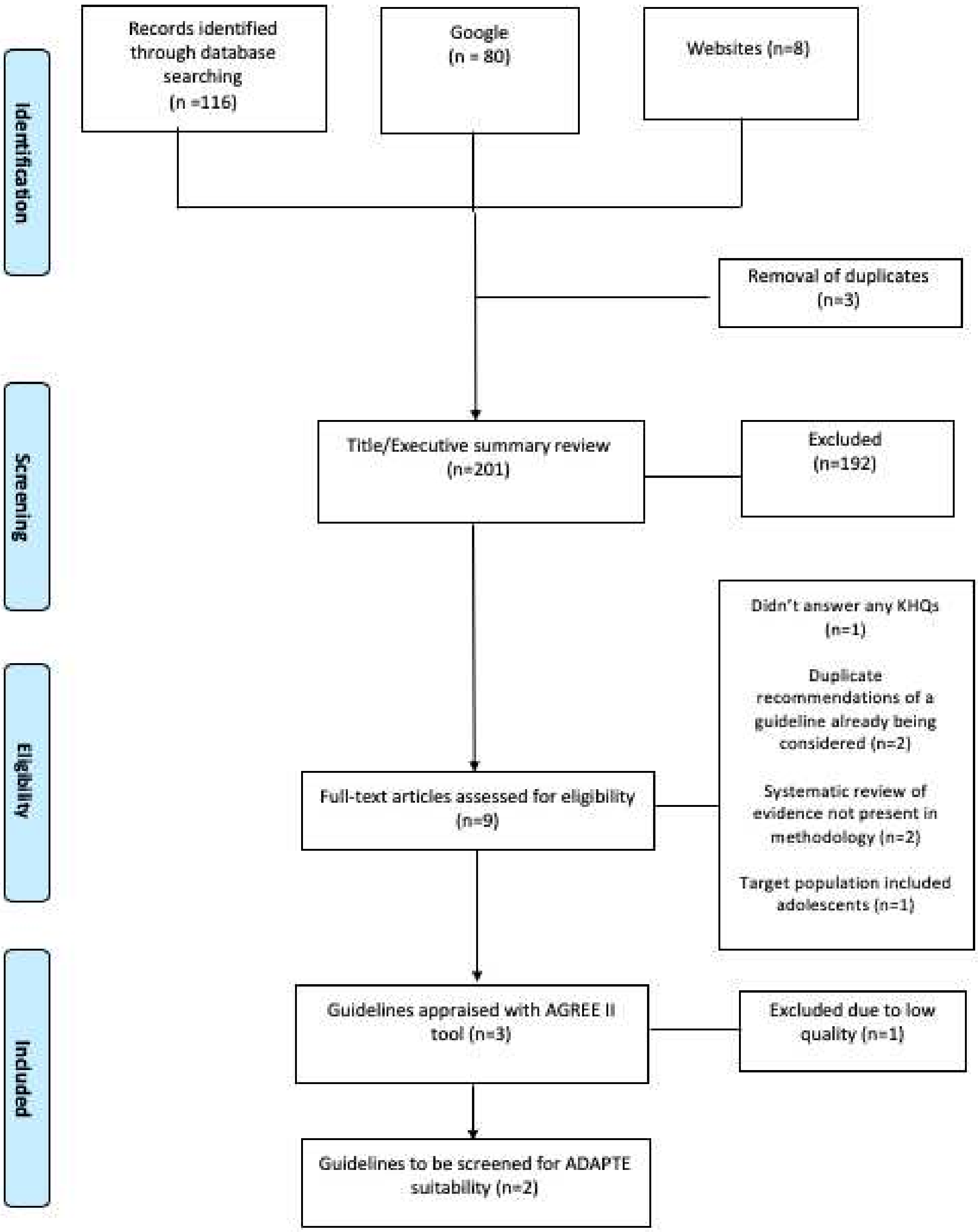

Figure 1 - Guideline review PRISMA flow diagram

Part E – AGREE 2 application to available guideline recommendations

Domain	BAP 2016 (%)[7]	WHO 2018 (%)[8]	SIGN 2013 (%)[11]
Scope and Purpose	56	89	89
Stakeholder involvement	38	67	78
Rigour of Development	25	86	47 [23]
Clarity of presentation	50	83	89
Applicability	17	52	25
Editorial independence	33	79	50
Overall assessment (whether the guideline should be considered for use in clinical practice)	**N**	**Y/M**	**Y/M**
ICC >0.9 across all domains. Inter-rater reliability was calculated using an intraclass correlation (two-way mixed-effects model) with SPSS version 26 (IBM Corp, Armonk, NY, USA).			

Table 2 - Domain scores for guidelines using AGREE II as assessed by two raters and scaled as a percentage of the maximum possible score. N=No, Y/M = Yes, with modifications, Y = Yes. Abbreviations: British Association of Psychopharmacology (BAP), Scottish Intercollegiate Guidelines Network (SIGN), World Health Organisaiton (WHO).

Part F – Part B empiric evidence review results

PubMed, PsycINFO and Embase search results

Search number	Search terms used	Hits
**1**	"Severe Mental Illness" OR schizophre* OR Psychosis OR Antipsychotic OR exp Psychotic Disorders OR First "Episode Psych*" AND Pharmac* OR Treatment OR Metformin OR Drug Therapy OR Manag* And Weight OR BMI OR Fat OR Manag* or Metabolic side effects OR Waist circumference	Embase = 17<br>PubMed = 60<br>PsycINFO = 205
		**Total screened =** 282<br>For further review = 7<br>Duplicates = 2<br>Non-relevant = 273
**2**	S1 but substitute antipsychotic with psychotropic* OR Neuroleptic OR Aripiprazole OR amisulpride OR asenapine OR Chlorpromazine OR Clozapine OR Fluphenazine OR Fluperazine OR Haloperidol OR Olanzapine OR Risperidone OR Paliperidone OR Quetiapine OR Zuclopenthixol	Embase = 66<br>PubMed = 517<br>PsycINFO = 423
		**Total screened** = 1006<br>For further review = 12<br>Duplicates = 23<br>Non-relevant = 971
**Identified through hand reference search = 6**		

BMJ Publishing Group Limited (BMJ) disclaims all liability and responsibility arising from any reliance placed on this supplemental material which has been supplied by the author(s)

Part G – PRISMA flow diagram of Literature review results (Part B)

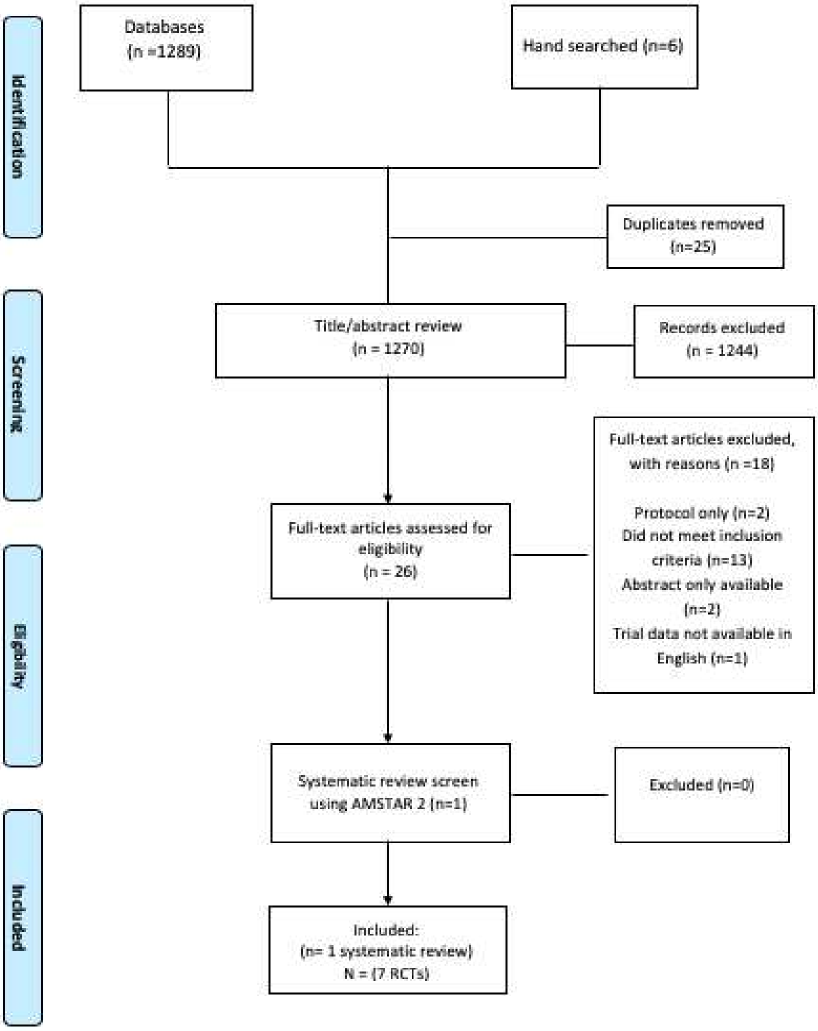

Figure 1 – Research evidence search PRISMA flow diagram

Fitzgerald I, *et al. Evid Based Ment Health* 2022; 25:15–22. doi: 10.1136/ebmental-2021-300291

Part H – Summary details of all Randomised Controlled Trials that met inclusion criteria (Part B)

	**Country**	**Methods**	**Number of Participants**	**Participants**	**Dose Metformin**	**Additional Interventions**	**Risk of bias – additionally see Appendix 2**
**Chiu 2016**	Taiwan	Parallel group RCT, 12 weeks	Metformin 500mg day (n=18), Metformin 1000mg/day (n=19), placebo (n=18)	Schizophrenia or schizoaffective disorder, aged 20-65 years	500mg/day and 1000mg/day in two separate arms	None	Low
**Chen 2012**	Taiwan	Parallel group RCT, 24 weeks	Placebo (n=27)	Schizophrenia or schizoaffective disorder taking clozapine > 3 months and were overweight or obese and fulfilled one or more criteria for metabolic syndrome, aged 20-65 years	1500mg/day	None	Unclear

**De Silva 2015**	Sri Lanka	Parallel group RCT, 24 weeks	Metformin (n=34)<br><br>Placebo (n=32)	Schizophrenia or schizoaffective disorder aged ≥ 18 years, treated with atypical antipsychotics who had an increase in pre-antipsychotic baseline body weight by ≥ 10%.	1000mg/day	Diet and lifestyle advice given at study commencement only	Low
**Jarskog 2013**	United States	Parallel group RCT, 16 weeks	Metformin (n=58), Placebo (n=58)	Schizophrenia or schizoaffective disorder, aged 18-65 years old, duration of illness ≥1 year, had a BMI ≥27, who were treated with one or a combination of two antipsychotics	2000mg/day	Weekly diet and exercise counselling	Low

**Wang 2012**	China	Parallel group RCT, 12 weeks	Metformin (n=32), Placebo (n=34)<br><br>Metformin + Lifestyle intervention (n=32)<br><br>Lifestyle intervention alone (n=32)	First episode schizophrenia, aged 18-60, gained ≥ 10% of their pre-drug body weight within the 1 year of treatment with clozapine, olanzapine, risperidone or sulpride	2000mg/day	None	Unclear
**Wu 2008 (a)**	China	Parallel group RCT, 12 weeks	Metformin (n=32)<br>Placebo (n=32)<br>Metformin + Lifestyle intervention (n=32)<br>Lifestyle intervention alone (n=32)	Aged 18-45, first episode schizophrenia, had gained ≥ 10% of their baseline body weight following treatment with sulpride, olanzapine, clozapine or risperidone	750mg/day	In the lifestyle intervention arm, a psychoeducational, dietary and exercise programme was provided. Psychoeducational groups were delivered at baseline and weeks 4, 8 and 12. A specific diet was provided for by a dietician. The lifestyle intervention involved 30-minute daily exercise sessions.	Low

**Wu 2012**	China	Parallel group RCT, 24 weeks	Metformin (n=42), Placebo (N=42)	Aged 18-40 years, first episode psychosis in female patients only	1000mg/day	None	Low

Part I – GRADE evidence profiles

I.1 Table 1 - Should Metformin vs. Usual Care or Placebo be used in the Treatment of AIWG in Adults with Established Psychosis?

Author(s):

1. De Silva et al,.
2. Chiu et al,.

Question: Should Metformin vs. Usual Care or Placebo be used in the Treatment of AIWG in Adults with Established Psychosis?
Setting: SU with schizophrenia or schizoaffective disorder
Follow up:
[1] = Mean duration 16 weeks
[2] = 12 weeks

Bibliography:

1. de Silva, V. et al. (2016). Metformin in prevention and treatment of antipsychotic induced weight gain: a systematic review and meta-analysis. *BMC Psychiatry*, [online] 16(1).
2. Chiu, C. et al. (2016). Effects of Low Dose Metformin on Metabolic Traits in Clozapine-Treated Schizophrenia Patients: An Exploratory Twelve-Week Randomized, Double-Blind, Placebo-Controlled Study. *PLOS ONE*, 11(12), p.e0168347.

PICO Key Health Questions Addressed:

KHQ 1 - Should metformin vs. usual care or placebo be used in the management of AIWG in adults with established psychosis?
KHQ 5 - Where metformin is identified as being effective in a particular cohort, what dose of metformin should be used?
KHQ 6 - Where metformin is identified as being effective in a particular cohort, for how long should metformin be used?
KHQ 7 - Where metformin is being used for the management of AIWG vs. usual care or placebo what are the potential harms associated with its use in adults with psychosis?

Certainty assessment	№ of patients	Effect	Certainty	Importance

№ of studies	Study design	Risk of bias	Inconsistency	Indirectness	Imprecision	Publication bias	Metformin	Vs. Usual Care or Placebo	Relative (95% CI)	Absolute (95% CI)		
**Change in weight (kg)**												
10 [1]	randomised trials	serious [a]	not serious	not serious	not serious	undetected	340	341	-	MD **3.24 kg lower** (4.72 lower to 1.76 lower)	⨁⨁⨁◯ MODERATE	CRITICAL
**Change in BMI (kg/m²)**												
10 [1]	randomised trials	serious [a]	not serious	not serious	not serious	undetected	340	341	-	MD **1.11 kg lower** (1.62 lower to 0.6 lower)	⨁⨁⨁◯ MODERATE	CRITICAL

Frequency of adverse events – narrative*

Certainty assessment							№ of patients		Effect		Certainty	Importance
№ of studies	Study design	Risk of bias	Inconsistency	Indirectness	Imprecision	Publication bias	Metformin	Vs. Usual Care or Placebo	Relative (95% CI)	Absolute (95% CI)		
5 [1]	randomised trials	serious [b]	serious [c]	not serious	not serious	undetected	**Discontinuation:** Only 5/10 studies reported on discontinuation (metformin, n=215; placebo, n=211). In all of these studies there was no significant difference in dropout rates across the two groups.<br><br>**Adverse events:** 8/10 studies reported adverse events. 6/8 studies (metformin = 227, placebo =223) reported whether there was a significant difference in adverse events between groups. Adverse events reported on varied across studies, and included most commonly dizziness, nausea and vomitting and diarrhea. Only in 1/6 was there a significance difference in adverse events - diarrhoea (metformin 33% vs. placebo 19%, p=0.018). No side-effects that aren't already common to metformin in other cohorts e.g. Type 2 diabetes mellitus, were reported. In the other 2 studies numerical imbalances were reported but it was unclear in the data whether differences were significant or whether same was assessed. No serious adverse events related to metformin treatment were reported.				⨁⨁◯◯ LOW	IMPORTANT

Change in weight (kg) – Metformin 1000mg/day**

Certainty assessment							№ of patients		Effect		Certainty	Importance
**№ of studies**	**Study design**	**Risk of bias**	**Inconsistency**	**Indirectness**	**Imprecision**	**Publication bias**	**Metformin**	**Vs. Usual Care or Placebo**	**Relative (95% CI)**	**Absolute (95% CI)**		
1 [2]	randomised trials	not serious	not serious	not serious	serious [d]	undetected	18	18	-	MD **1kg lower** (CI not reported) in the metformin compared to placebo group	⊕⊕⊕◯ MODERATE	CRITICAL
**Change in BMI (kg/m²) - Metformin 1000mg/day****												
1 [2]	randomised trials	not serious	not serious	not serious	serious [d]	undetected	18	18	-	MD **0.5 lower** (CI not reported) in the metformin compared to placebo group	⊕⊕⊕◯ MODERATE	CRITICAL
**Frequency of adverse events – narrative****												
1 [2]	randomised trials	not serious	not serious	not serious	not serious	undetected	No serious adverse events were reported. Incidence of side-effects weren't significantly different among the three groups.				⊕⊕⊕⊕ HIGH	IMPORTANT

CI: Confidence interval; **MD:** Mean difference; **ET:** End of Trial

* Adverse event data was not synthesized quantitatively for several reasons. This included evidence of incomplete and selective outcome reporting in some studies meaning that data were incomplete. As highlighted in 8/10 studies in the De Silva et al., meta-analysis did report adverse event data, however in only 6/8 cases numerical data (total study numbers: placebo n =223, metformin n =227) was reported that could be considered for aggregation. Furthermore, a range of side effects were reported with very small number of events per subgrouping e.g. within GI side effects, some studies reported on nausea, others on diarrhoea only. In other cases a range of seemingly unrelated side effects were reported including somnolence and extrapyramidal s/e, which haven't been related to metformin treatment previously. One study only gave adverse event data where the rate of that adverse event that affected >5% of the overall sample. We received no response from study authors for greater details where the study report did not provide numerical data, or where the report stated that there was no significant difference in adverse events, but did not report the adverse effects measured or number of associated events. Due to missing data, the sample size of available studies where data was available to combine, and smaller number of events within these subcategories of side effects – even amongst s/e that are known to be common to metformin i.e. gastrointestinal side effects, we decided that pooling these results was unlikely to yield clinically meaningful results due to low power and precision of any estimate drawn, and that certainty in collated estimates would be very low. We agreed quantitative aggregation wouldn't yield any further benefits over narrative synthesis and thus, wouldn't influence our application of the evidence base specific to this outcome and resultant recommendations.

** Results for this study were assessed separately to the meta-analysis by De Silva et al.[1] The guideline development group chose to do this primairly because the population under assessment in the RCT by Chiu et al.,were those treated with clozapine i.e. participants were those with treatment-resistant schizophrenia. The mean duration of illness was 25-28 years across treatment groups and thus previous antipsychotic exposure was very likely significant, although not specified in the study report.[2] As the effects of metformin in this group compared to those who have a much lesser burden of antipsychotic exposure are likely to be different, as highlighted in the De Silva et al., sub-group analysis,[1] a decision was made not to collate these results. Furthermore, doses used were significantly lower than those applied across studies included in the De Silva et al., meta-analysis.[1,2] As the study report by Chiu et al., did not provide any measure of variance of effect alongside point estimates,[2] this represented another barrier to aggregation of study results. Requests for such measures from the study authors by the guideline development group went unanswered.

a. = Rated serious as there was an unclear risk of bias for blinding in four studies and an unclear risk of bias related to drop-out rates in one. Despite anthropometric outcomes being objective, it was felt that if participants, research personnel and/or outcome assessors were aware of individual(s) assignment, this may lead to an alteration in health behaviours and reduce certainty associated with estimates.

b = Marked as serious due to high risk of bias in 2/10 studies – due to no reporting on this outcome i.e. selective outcome reporting. In 2/8 that did, this data was incomplete i.e. evidence of incomplete outcome reporting.

c = Although data wasn't pooled, results appeared inconsistent. This was worsened by incomplete and selective outcome reporting in minority of studies.

d = Downgraded as no confidence intervals were reported for any of the outcomes assessed.

Fitzgerald I, *et al. Evid Based Ment Health* 2022; 25:15–22. doi: 10.1136/ebmental-2021-300291

I.2 Table 2 – Should Metformin vs. Non-Pharmacological Treatment be used in the Management of AIWG with Psychosis?

Author(s): Wu et al,.
Question: Should Metformin vs. Non-Pharmacological Treatment be used in the Management of AIWG with Psychosis?
Setting: SU with schizophrenia or schizoaffective disorder
Follow up: 12 weeks

Bibliography:
1. Wu, R. et al. (2008). Lifestyle intervention and metformin for treatment of antipsychotic-induced weight gain: a randomized controlled trial. JAMA, [online] 299(2), pp.185-193.

PICO Key Health Questions Addressed:

KHQ 2 – Should Metformin vs. Non-pharmacological treatment be used in the management of AIWG in adults with psychosis?
KHQ 5 - Where metformin is identified as being effective in a particular cohort, what dose of metformin should be used?
KHQ 6 - Where metformin is identified as being effective in a particular cohort, for how long should metformin be used?
KHQ 7 - Where metformin is being used for the management of AIWG vs. usual care or placebo what are the potential harms associated with its use in adults with psychosis?

Certainty assessment							№ of patients		Effect		Certainty	Importance
№ of studies	Study design	Risk of bias	Inconsistency	Indirectness	Imprecision	Publication bias	Metformin	Non-Pharmacological Treatment	Metformin Absolute (95% CI)	Non-Pharmacological Absolute (95% CI)		
**Mean change in weight (kg)**												
1	randomised trials	not serious	not applicable	serious [a]	not serious	undetected	32	32	MD **3.2kg lower** (3.9 to 2.4 lower)	MD **1.4 kg lower** (2.0 lower to 0.7 lower)	⨁⨁⨁◯ MODERATE	CRITICAL

Mean change in BMI (kg/m²)

1	randomised trials	not serious	not applicable	serious [a]	not serious	undetected	32	32	MD **1.2 lower** (1.5 lower to 0.9 lower)	MD **0.5 lower** (0.8 to 0.3 lower)	⨁⨁⨁◯ MODERATE	CRITICAL

Frequency of adverse events – narrative

1	randomised trials	not serious	not applicable	serious [a]	not serious	undetected	There was no statistically significant difference between any type of adverse event seen in the two groups. Numerically higher rates of nausea was seen in the metformin treated groups. There were five serious adverse events in the trial that led to withdrawal. All were exacerbation of psychosis.	⨁⨁⨁◯ MODERATE	IMPORTANT

CI: Confidence interval; **MD:** Mean difference

a. Rated down by one level as the included cohort were FEP participants who had gained >10% of their baseline bodyweight and in all groups' total exposure to antipsychotics was < 1 year. Thus, this cohort are only representative of some of the SU that will be affected by guideline recommendations i.e. generalisability reduced.

I.3 Table 3 - Should Metformin + Non-Pharmacological treatment versus Non-Pharmacological treatment alone be used in the Management of AIWGs in Adults with Psychosis?

Author(s): Wu et al,.
Question:Should Metformin + Non-Pharmacological treatment versus Non-Pharmacological treatment alone be used in the Management of AIWGs in Adults with Psychosis?
Setting: SU with schizophrenia or schizoaffective disorder
Follow up: 12 weeks

Bibliography:
1. . Wu, R. et al. (2008). Lifestyle intervention and metformin for treatment of antipsychotic-induced weight gain: a randomized controlled trial. JAMA, [online] 299(2), pp.185-193.

PICO Key Health Questions Addressed:

KHQ 3 - Should metformin + non-pharmacological vs. non-pharmacological treatment alone be used in the management of AWIG in adults with psychosis?
KHQ 5 - Where metformin is identified as being effective in a particular cohort, what dose of metformin should be used?
KHQ 6 - Where metformin is identified as being effective in a particular cohort, for how long should metformin be used?
KHQ 7 - Where metformin is being used for the management of AIWG vs. usual care or placebo what are the potential harms associated with its use in adults with psychosis?

Certainty assessment							№ of patients		Effect		Certainty	Importance
№ of studies	Study design	Risk of bias	Inconsistency	Indirectness	Imprecision	Publication bias	Metformin + Non-Pharmacological Management	Non-Pharmacological Management	Metformin + Non-Pharmacological Management – Absolute (95% CI)	Non-Pharmacological Management-Absolute (95% CI)		

Change in weight (kg)

Fitzgerald I, *et al. Evid Based Ment Health* 2022; 25:15–22. doi: 10.1136/ebmental-2021-300291

Certainty assessment							№ of patients		Effect		Certainty	Importance
№ of studies	Study design	Risk of bias	Inconsistency	Indirectness	Imprecision	Publication bias	Metformin + Non-Pharmacological Management	Non-Pharmacological Management	Metformin + Non-Pharmacological Management – Absolute (95% CI)	Non-Pharmacological Management- Absolute (95% CI)		
1	randomised trials	not serious	not applicable	serious [a]	not serious	undetected	32	32	MD **4.7 kg lower** (5.7 lower to 3.4 lower)	MD **1.4 lower** (2.0 lower to 0.7 lower)	⨁⨁⨁◯ MODERATE	CRITICAL

Change in BMI (kg/m²)

1	randomised trials	not serious	not applicable	serious [a]	not serious	undetected	32	32	MD **1.8 lower** (2.3 to 1.3 lower)	MD **0.5 lower** (0.8 to 0.3 lower)	⨁⨁⨁◯ MODERATE	CRITICAL

Change in waist circumference (cm)

1	randomised trials	not serious	not applicable	serious[a]	not serious	undetected	32	32	mean **2.0 lower** (2.4 to 1.5 lower)	mean **0.1 higher** (0.5 lower to 0.7 higher)	⨁⨁⨁◯ MODERATE	CRITICAL

Frequency of adverse events – narrative

Fitzgerald I, *et al. Evid Based Ment Health* 2022; 25:15–22. doi: 10.1136/ebmental-2021-300291

Certainty assessment							№ of patients		Effect		Certainty	Importance
№ of studies	Study design	Risk of bias	Inconsistency	Indirectness	Imprecision	Publication bias	Metformin + Non-Pharmacological Management	Non-Pharmacological Management	Metformin + Non-Pharmacological Management – Absolute (95% CI)	Non-Pharmacological Management-Absolute (95% CI)		
1	randomised trials	not serious	not applicable	serious [a]	not serious	undetected	32	32	Rates of adverse gastrointestinal events were 12.5% in the metformin + lifestyle group and 15.6% in the lifestyle + placebo group, with no significant difference between groups (P<0.88). No difference in dropouts between groups due to adverse GI events. Notably in this study the maximum dose of metformin used was 750mg/day. This dose combined with the small numbers in each group may have led to the lack of significance between groups, as this is a common significant side effect of metformin in groups with bigger numbers.		⊕⊕⊕◯ MODERATE	IMPORTANT

CI: Confidence interval; **MD:** Mean difference

Footnotes:

a = Rated down by one level as the included cohort were FEP participants who had gained >10% of their baseline bodyweight and in all groups' total exposure to antipsychotics was < 1 year. Thus, this cohort are only representative of some of the SU that will be affected by guideline recommendations i.e. generalisability reduced.

I.4 Table 4 - Should Metformin compared to Placebo or Usual Care be Used in the Management of AIWG in FEP?

Author(s): De Silva et al,.
Question: Should Metformin compared to Placebo or Usual Care be Used in the Management of AIWG in **FEP?**
Setting: SU with FEP
Follow up: Mean follow up 14 weeks

Bibliography:

1. de Silva, V. et al. (2016). Metformin in prevention and treatment of antipsychotic induced weight gain: a systematic review and meta-analysis. *BMC Psychiatry*, [online] 16(1).

PICO Key Health Questions Addressed:
KHQ 4 - Should metformin vs. usual care or placebo be used in the management of AWIG in adults with **FEP?**
KHQ 5 - Where metformin is identified as being effective in a particular cohort, what dose of metformin should be used?
KHQ 6 - Where metformin is identified as being effective in a particular cohort, for how long should metformin be used for?

Certainty assessment							№ of patients		Effect		Certainty	Importance
№ of studies	Study design	Risk of bias	Inconsistency	Indirectness	Imprecision	Publication bias	Metformin	Placebo or Usual Care	Relative (95% CI)	Absolute (95% CI)		
**Mean change in weight (kg)**												
5	randomised trials	serious [a]	not serious	not serious	not serious	undetected	140	143	-	MD **5.94 lower** (6.75 lower to 5.12 lower)	⊕⊕⊕◯ MODERATE	CRITICAL

Fitzgerald I, *et al. Evid Based Ment Health* 2022; 25:15–22. doi: 10.1136/ebmental-2021-300291

CI: Confidence interval; **MD:** Mean difference

a. Downgraded by one level for two reasons:
(1) Evidence of high risk of bias due to in selective outcome reporting in 1/5 studies.
(2) Unclear risk of bias in 2/5 studies across RoB outcomes related to sequence generation for randomization, allocation sequence concealment and blinding of participants and personnel.
